# Comparison of Whole Genome Amplification Methods for Analysis of DNA Extracted from Microdissected Early Breast Lesions in Formalin-Fixed Paraffin-Embedded Tissue

**DOI:** 10.5402/2012/710692

**Published:** 2012-03-14

**Authors:** Nona Arneson, Juan Moreno, Vladimir Iakovlev, Arezou Ghazani, Keisha Warren, David McCready, Igor Jurisica, Susan J. Done

**Affiliations:** ^1^Division of Applied Molecular Oncology, Ontario Cancer Institute, Princess Margaret Hospital, Toronto, ON, Canada M5G 2M9; ^2^Campbell Family Institute for Breast Cancer Research, Toronto, ON, Canada M5G 2M9; ^3^Laboratory Medicine Program, University Health Network, Toronto, ON, Canada M5G 2C4; ^4^Department of Laboratory Medicine, St. Michael's Hospital, Toronto, ON, Canada M5B 1W8; ^5^Department of Laboratory Medicine and Pathobiology, University of Toronto, Toronto, ON, Canada M5S 1A8; ^6^Department of Surgical Oncology, Princess Margaret Hospital, Toronto, ON, Canada M5G 2C4; ^7^Campbell Family Institute for Cancer Research, Princess Margaret Hospital, Techna Institute, University Health Network, Toronto, ON, Canada M5G 2C1; ^8^Department of Medical Biophysics, University of Toronto, Toronto, ON, Canada M5G 1A1; ^9^Department of Computer Science, University of Toronto, Toronto, ON, Canada M5S 3G4

## Abstract

To understand cancer progression, it is desirable to study the earliest stages of its development, which are often microscopic lesions. Array comparative genomic hybridization (aCGH) is a valuable high-throughput molecular approach for discovering DNA copy number changes; however, it requires a relatively large amount of DNA, which is difficult to obtain from microdissected lesions. Whole genome amplification (WGA) methods were developed to increase DNA quantity; however their reproducibility, fidelity, and suitability for formalin-fixed paraffin-embedded (FFPE) samples are questioned. Using aCGH analysis, we compared two widely used approaches for WGA: single cell comparative genomic hybridization protocol (SCOMP) and degenerate oligonucleotide primed PCR (DOP-PCR). Cancer cell line and microdissected FFPE breast cancer DNA samples were amplified by the two WGA methods and subjected to aCGH. The genomic profiles of amplified DNA were compared with those of non-amplified controls by four analytic methods and validated by quantitative PCR (Q-PCR). We found that SCOMP-amplified samples had close similarity to non-amplified controls with concordance rates close to those of reference tests, while DOP-amplified samples had a statistically significant amount of changes. SCOMP is able to amplify small amounts of DNA extracted from FFPE samples and provides quality of aCGH data similar to non-amplified samples.

## 1. Introduction

Early breast cancer detection, accurate diagnosis, and personalized therapies have become goals of clinical medicine in the genomic era, and in the last few decades molecular genetics advances have contributed greatly to the development of those goals. The ability to identify genomic characteristics, determine copy number variations, or measure RNA and miRNA with a variety of technologies provided the medical field with tools to explore the molecular make up of any sample and compare physiologic and pathologic states from any human tissue. However, the accuracy of such techniques depends largely on the purity of the samples provided for analysis. At the same time, a relatively large amount of nucleic acid is required for accurate results. Microdissection techniques have increased the purity of samples, enabled us to study the earliest stages of disease development, and allow separation of different tissue constituents, for example, separating epithelial tissue from the surrounding stroma in a breast lesion. A consequence of increased targeting ability is a corresponding decrease in the amount of nucleic acid available for research. In the breast cancer research field, examples of areas that deal with small lesions and limited samples are the study of breast carcinoma *in situ* which is a presumed precursor to invasive breast carcinoma and a growing clinical problem [1, 2] and establishing the role of the stroma or myoepithelial cells in the development of pre-invasive and invasive lesions. Some breast lesions like atypical ductal hyperplasia and flat epithelial atypia are of much interest to pathologists and clinicians as they are upgraded to carcinoma about 10–20% of the time in subsequent studies; however opportunities to study such lesions are limited because they are often discovered incidentally in biopsies, and therefore the amount of samples available for research is very small.

In breast cancer research, correlation of molecular characteristics with outcomes helps identify predictive and prognostic variables that are of great value in clinical practice, unfortunately, most of the time that information can only be obtained after lengthy periods of followup to record whether or not the outcome of interest develops. This issue makes finding prognostic and predictive markers very costly and time consuming. Conversely, there are millions of FFPE samples worldwide, many associated with detailed clinical data that makes them a precious resource for survival studies and prognostic and predictive marker development. Therefore, technologies that can be used on FFPE tissues without being limited by the amount of sample are very valuable.

Array CGH (aCGH) is being widely used to identify the areas of genomic gain and loss that occur in different types of lesions and offers high-throughput capability, high resolution, and precise mapping of aberrations [3–6]. Several microarray platforms have been developed and used for aCGH including cDNA arrays [7–9], oligonucleotide arrays [10, 11], BAC arrays [12–17], and most recently SNP arrays [18–21]. Unfortunately, in order to be useful in the molecular pathology lab, this type of molecular analysis requires an abundant supply of high-quality genomic DNA from clinical specimens, not only for the array CGH analysis but also to validate results using an independent technique such as Q-PCR.

 To facilitate molecular analysis of small specimens, several methods of whole genome amplification have been developed [22]. A commonly used method is degenerate oligonucleotide primed PCR (DOP-PCR) [23]. DOP-PCR has been modified and used for several different types of molecular analysis including chromosomal CGH [24–28], high-resolution (HR-) CGH [29, 30], genotyping [31, 32], LOH analysis [33], mutation detection [34], array CGH [35–37] and more recently for methylation profiling of trace amounts of DNA [38, 39]. Although DOP-PCR has been widely accepted as a method of whole genome amplification, it is known that it introduces amplification bias. While some have been successful using DOP-PCR on FFPE tissues [40], others have found that DOP-PCR is not well suited for archived specimen analysis [41]. Artifactual amplification at chromosomes 1p, 3, 13q, and 16p as well as preferential amplification of shorter alleles [37] has been reported. Grant et al. consider DOP-PCR a useful amplification method if researchers monitor carefully storage conditions and accept a “modest increase” in genotyping error [42]. Multiple displacement amplification (MDA) [43] and modifications of MDA such as restriction- and circularization-aided rolling circle amplification (RCA-RCA) [44] and MDA using the large fragment of Bst DNA polymerase [45] have been used on FFPE tissue as a method of WGA for aCGH [46–48]. The disadvantage of these methods, which employ a polymerase, is that they may not perform well with degraded DNA extracted from FFPE tissues [46], and the efficiency and accuracy of MDA vary with the cell type [49]. A recent review on the MDA method using nonfixed samples reports that it introduced pronounced skewing when evaluating ribosomal RNA [50]. To deal with MDA bias, some have suggested combining two MDA reactions, one denatured and one nondenatured, aiding copy number analysis and subsequent genotyping [51].

The methods based on producing representative amplicons by ligation-mediated PCR (LM-PCR) [52, 53], balanced PCR amplification, and adaptor-ligation PCR of randomly sheared genomic DNA (PRSG) have all performed well for array CGH [44, 54, 55] where the random-primed amplification (RPA) has been used successfully with FFPE tissues for array CGH and found to be superior to degenerate oligonucleotide-primed amplification for array-based CGH [56].

 Other technologies recently marketed for whole genome amplification include a linker-adapted PCR-based proprietary kit that was shown to be superior to MDA, DOP-PCR, random priming, and RCA-RCA methods for FFPE samples [57], OmniPlex which reports good results on WGA of FFPE tissue prior to SNP analysis [58] and repli-G which is marketed as a WGA kit that uses the previously known Phi29 DNA polymerase method and adds a ligation step prior to amplification [27].

In 1999, Klein et al. published a ligation-mediated method of whole genome amplification paired with chromosomal CGH that was specifically designed for the analysis of genomes of single cells and was termed “SCOMP” (single-cell comparative genomic hybridization) [59].This method has been used successfully for CGH analysis of FFPE specimens [41, 60] and for CGH analysis of single cells [61, 62]. SCOMP was found to be superior to DOP-PCR for global amplification of very small amounts of DNA from microdissected FFPE samples [41].

While several groups have demonstrated that it is possible to perform WGA on FFPE samples, few have critically assessed the resulting DNA for reproducibility and fidelity of replication on a genome-wide scale. We have tested several methods of WGA, systematically analyzed their performance, and selected the two best performing, SCOMP and DOP-PCR, for further assessment. The degree of WGA effect on identification of genomic alterations was quantified and compared between the methods. This assessment is a necessary validation step of WGA methods and, we believe, provides invaluable information for scientists using FFPE samples for aCGH studies.

### 1.1. Samples and DNA Extraction

20 *μ*g of genomic DNA from the UACC-812 breast cancer cell line (ATCC, http://www.atcc.org/) was extracted using the QIAmp DNA Mini Kit (Qiagen, Canada) according to the manufacturer's instructions. DNA from the cell line and human placenta was digested with 2.5 U each of RsaI and AluI restriction enzymes (Invitrogen) in a final volume of 100 *μ*L. The digested DNA ranging in size from 100 to 10,000 base pairs was cleaned using the Qiaquick PCR Purification Kit (Qiagen, Canada) and quantitated using a DyNA Quant Fluorometer (Amersham Biosciences).

For FFPE samples, multiple 5 *μ*m thick sections of paraffin blocks were deparaffinized and stained for 30 sec in haematoxylin prior to microdissection. Tumor areas were isolated in a dissecting stereo microscope using 18 G needles and H&E-stained slides for guidance (under supervision of a pathologist, S. J. Done). Microdissected tissue was incubated in lysis buffer for 72 hr, and DNA was extracted using the QIAmp DNA Mini Kit. 

### 1.2. DOP-PCR

Degenerate oligonucleotide primed (DOP-) PCR was performed by two methods. First, using the DOP-PCR Master Kit (Roche) according to the manufacturer's instructions; second, according to the protocol of Huang et al. [27]. Briefly, 10–100 ng of genomic DNA was amplified by Thermo Sequenase (Amersham Biosciences) in a low-stringency preamplification step (5 cycles), followed by regular PCR amplification in less stringent conditions. The fragments generated by both methods ranged from 100 to 1000 base pairs. For both methods, several replicate reactions were pooled together (*n* = 5–7 based on the sample size, i.e, amount of material available) and a negative control (template: water) was used to ensure absence of contamination. The resulting amplified DNA was purified using the Qiaquick PCR Purification Kit (Qiagen, Canada) and quantified using a DyNA Quant Fluorometer (Amersham Biosciences).

### 1.3. SCOMP

The initial steps of the SCOMP procedure were performed according to the protocol provided by Dr. Klein, starting with 30–150 ng of template DNA. Genomic DNA was digested with 2 U MseI (New England Biolabs) for 3 hours in One-Phor-All Buffer (Amersham) in a final volume of 5 *μ*L. Base pairing of the adaptor nucleotides was done in a final volume of 3 *μ*L using 0.5 *μ*L One-Phor-All Buffer, 0.5 *μ* 100 *μ*M Lib1 oligonucleotide (5′-AGTGGGATTCCTGCTGTCAGT-3′), and 0.5 *μ*L 100 *μ*M ddMse11 oligonucleotide (5′-TAACTGACAGCdd-3′) in a MJ Research PT100 thermocycler programmed for a gradient of 65°C to 15°C ramped down at 1°C per minute. 1 *μ*L T4 DNA Ligase (40 U/*μ*L) (Roche), 1 *μ*L of 10 mM ATP and the MseI digested genomic DNA was added and allowed to incubate at 15°C overnight. For PCR amplification the following was added to the ligation mix: 3 *μ*L Expand Long Template buffer 1 (Roche), 2 *μ*L 10 mM dNTPs, 35 *μ*L H_2_O and 1 *μ*L Expand-Long-Template PolMix (3.5 U/*μ*L). Thermocycler conditions were as follows: 1 cycle, 68°C for 3 mins; 15 cycles 40 sec at 94°C, 30 sec at 57°C, 1 min 30 sec + 1 sec/cycle at 68°C; 8 cycles 40 sec at 94°C, 30 sec at 57°C + 1°C/cycle, 1 min 45 sec + 1 sec/cycle at 68°C; 22 cycles 40 sec at 94°C, 30 sec at 65°C, 1 min 53 sec + 1 sec/cycle at 68°C; 1 cycle 3 min 40 sec at 68°C. Several reactions were pooled together (*n* = 5–7 as above). A negative control (template: water) was used for all steps to ensure there was no contamination. Following PCR amplification, the resulting products were cleaned with the Qiaquick PCR Purification Kit (Qiagen, Canada) and quantitated using a DyNA Quant Fluorometer (Amersham Biosciences).

### 1.4. Array CGH

2-3 *μ*g of DNA was labelled by random priming (Bioprime DNA labeling Kit, Invitrogen) in 3 separate reactions with either Cy3 or Cy5. Labeled products were mixed in appropriate combinations in DIG Easy Hyb (Roche) hybridization buffer and hybridized for 16–24 hours at 37°C in a humidified chamber in duplicate to the Human 19 K single-spot cDNA arrays from the Clinical Genomics Centre, UHN (University Health Network Microarray Centre, http://www.microarrays.ca/), which contain 19,008 human ESTs/genes with map positions identified for ~11,000 cDNA clones with the median distance between mapped positions 73.4 Kb, where 93% of the clones spaced <1Mb and 99% <3Mb. Slides were rinsed and then washed 3 times and centrifuged to dry.

Arrays were scanned using the GenePix 4000A scanner (Axon Instruments, USA). The photomultiplier gain for each laser was adjusted to give an average ratio of Cy3 to Cy5 of 1 and to minimize the number of saturated pixels. Images were then analyzed using the GenePix Pro 3.0 software (Axon Instruments, USA). Each subgrid on each array was independently normalized by equalizing the Cy3 intensities with respect to the Cy5 intensities, while excluding spots flagged as anomalous or absent by the quantifying software. Log2 ratios were assigned to each spot and the profiles were centered by the median value and scale normalized by the median of absolute values.

### 1.5. Quantitative Real-Time (Q-RT) PCR

Primers were designed using the Primer Express software (Applied Biosystems). PCR was performed according to the ABI7700 protocols using the Quantitect SYBR Green PCR Kit (Qiagen, Canada). All PCR reactions were done in triplicate. DNA from normal placenta was used as reference and relative gene quantity was calculated by the delta-delta Ct method.

### 1.6. aCGH Analysis

**Figure 1:**
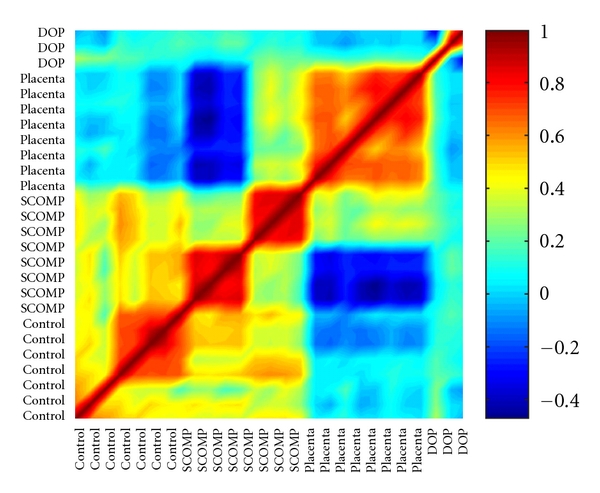
Pseudocolor representation of a correlation matrix, showing a relationship between nonamplified DNA (*n* = 8), SCOMP (SCOMP amplified, *n* = 8), placenta (self-hybridizations of normal genomic DNA, *n* = 8), and DOP (DOP-PCR amplified, *n* = 3) array experiments. The color map corresponds to the scale of correlation coefficients; positive correlated data range in color from light blue to dark red, negatively correlated data range from light blue to dark blue. The diagonal of the symmetric correlation matrix represents self-correlation and thus is equal to one (dark red).

**Figure 2:**
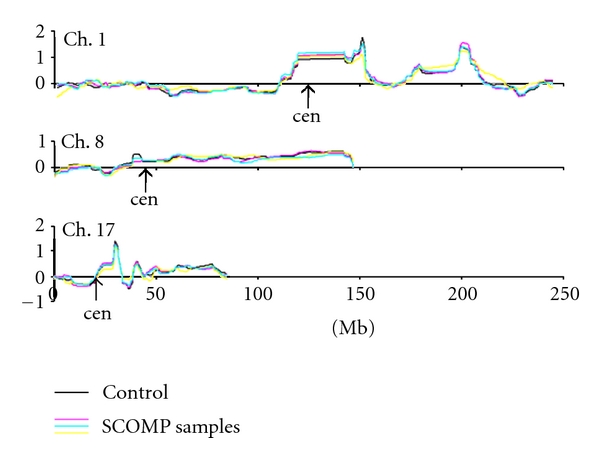
Examples of genomic alterations identified in UACC-812 cells: gains on 1q, 8q, 17q and losses on 1p and 17p. Note that SCOMP amplified samples have profiles almost identical to each other and to nonamplified control DNA.

First, we tested WGA methods using higher-quality DNA from the UACC-812 cell line and human placenta. Three independent runs of DNA from the cell line amplified by SCOMP and DOP methods were compared by correlation with nonamplified controls (Pearson). Since SCOMP was giving consistently better results, an additional five samples amplified by this technique were tested. The data was summarized by a pseudocolor matrix generated using the Matlab R12 (MathWorks Inc, Natick, MA, USA) software to display pair-wise correlations among individual samples (Figure 1). Significance analysis of microarrays (SAM; http://www-stat.stanford.edu/~tibs/SAM/) [63] was used to identify significantly amplified or deleted genes among the amplified and nonamplified datasets. A binary tree-structured vector quantization (BTSVQ; available at http://www.cs.toronto.edu/~juris/btsvq/downloads.html) algorithm was used for unsupervised cluster analysis. BTSVQ combines a partitive k-means clustering and a self-organizing maps (SOMs) algorithm in a complementary way, to achieve clustering of both samples and genes. A moving average of microarray data (sliding window of 20 data points, Figure 2) was used to search for genomic alterations previously reported in UACC-812 cells.

**Figure 3:**
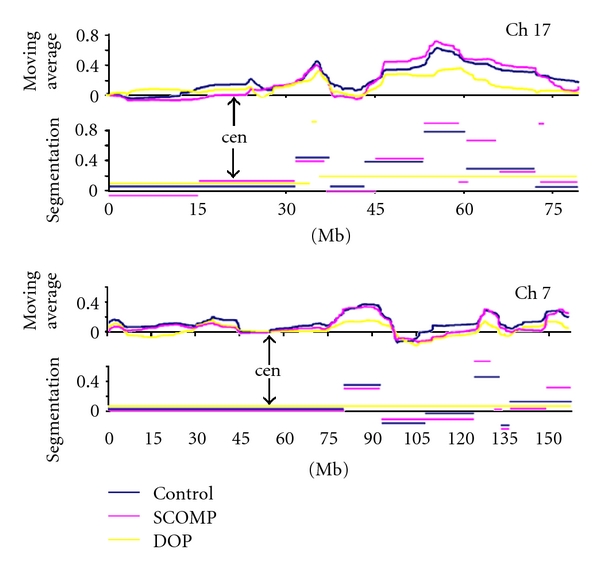
Example of aCGH analysis of FFPE breast cancer samples. Chromosomes 17 (upper panel) and 7 (lower panel) analyzed by moving average (upper graphs of both Ch17 and Ch7 panels) and segmentation (lower graphs). Ch17 of this sample showed amplifications at 17q12-21 centered at the HER2/neu gene, a broad region of gain on distal 17q, three regions of gain and one region of loss on 7q. SCOMP amplified samples resolved all segments and peaks identified in nonamplified control DNA, while DOP amplified samples had significant alterations of the profiles.

Our next step was to test WGA methods on FFPE microdissected breast cancer samples. Initially, three samples were amplified by SCOMP and DOP methods and compared to nonamplified controls. Then, since SCOMP performed better, three additional samples amplified by SCOMP were added to the analysis. All profiles were analyzed by Pearsons correlation of nonsegmented aCGH data between amplified samples and corresponding nonamplified controls. We also counted the number of individual cDNA spots concordant as gain or loss between the test and corresponding control samples as well as between the duplicate runs of nonamplified control DNA. Additionally, the profiles were analyzed as whole genomes by arranging clones in the genomic sequence (National Center for Biotechnology Information, build 201 and University of California, Santa Cruz, build hg18) and identifying genomic alterations by the circular binary segmentation algorithm (“DNAcopy” package ver. 2.5.0 for “R” ver. 2.5.0, http://www.r-project.org/) and peaks of moving average (Figure 3). Duplicate runs of nonamplified control samples were used to establish reference values for each type of analysis.

The segmentation algorithm was used to identify contiguous segments of statistically uniform individual data points. The segments of amplified samples and the segments of nonamplified controls were screened to identify regions of overlapping concordant gains and losses. The lengths of the concordant regions were summed, and the percentage of concordantly identified genome length was calculated for each amplified sample. Then, peaks of genomic alterations were visualized by moving average as described above, and the peaks of amplified samples of the same sign and position as the peaks of control samples were recorded as concordant without restrictions for amplitude. All other peaks were recorded as discordant. Percentages of concordant/discordant peaks were calculated per amplified sample. 

### 1.7. WGA

We have evaluated four major PCR-based methods of WGA and investigated their suitability for processing FFPE clinical samples. The methods include two variations of DOP-PCR [23, 27], SCOMP [41, 59], and T7-based linear amplification of DNA (TLAD) [64]. We did not investigate methods based on multiple displacement amplification (MDA) using DNA polymerase [43, 45, 46], because the enzyme may not be suitable for FFPE clinical samples due to fragmentation of the template genomic DNA [46]. Our preliminary tests eliminated two of the four methods. The DOP-PCR Master Kit (Roche, Mannheim, Germany) performed well with good-quality genomic DNA from our cell line, however, failed to amplify genomic DNA extracted from FFPE tissue. In addition, it was often subject to contamination in the negative control, which was of significant concern given the low quantities of genomic DNA we expected to use (as low as 10 ng). The TLAD protocol was laborious and costly, and there was significant loss of template DNA in the initial steps due to many purification steps. Although TLAD did significantly amplify the amount of starting material up to 100x in our hands, the CGH arrays failed due to what appeared to be poor labeling efficiency of the resulting products. The remaining DOP-PCR protocol and SCOMP performed well during preliminary tests and were further analyzed by aCGH. 

Both modified DOP-PCR and SCOMP performed equally well at amplifying genomic DNA from cell lines as well as from FFPE material. Starting with as little as 10 ng of genomic DNA, we were able to get as much as 2-3 *μ*g from the modified DOP-PCR protocol and SCOMP with products ranging in size from 10 to 10,000 bp, and 100 to 1,500 bp respectively. The negative controls (template: water) in both cases showed little or no product formation. When product formation was detected in the negative control lane, the products from that experiment were not used for analysis.

### 1.8. DNA from Cell Line and Placenta

UACC-812 cell line was used as a source of high-quality DNA. The cells have been shown to harbor several regions of amplification including 1q, 8q, 13q, 17q, and 20q [65] and amplification of the DNA topoisomerase II (TOP2) gene [66]. Our aCGH profiles of control samples showed alterations of the expected regions, including those on chromosomes 1, 8, and 17 shown in Figure 2.

To validate the microarray data by an independent molecular method, we performed Q-PCR on 15 genes found to be amplified by corresponding cDNA clones in amplified or nonamplified material, as well as 7 random genes. The relative ratio of UACC-812 to placenta was calculated for cDNA clones of aCGH and compared to relative gene quantity determined by Q-PCR. For nonamplified DNA, loss/gain was confirmed in 18 out of 22 samples with correlation of absolute values *r* = 0.62 (Pearson), for SCOMP-amplified in 19/22 (*r* = 0.75), and 9/22 (*r* = 0.22) in the DOP-amplified experiments (Table 1).

Initially, we tested and compared DOP and SCOMP methods using three samples in a group and then expanded the number of SCOMP amplified samples to further test the technique as it showed greater fidelity. Figure 1 shows pair-wise correlations between aCGH profiles. The highest reproducibility was observed for nonamplified samples. SCOMP-amplified samples showed higher correlation with the nonamplified controls than DOP-amplified samples. As expected, the self/self hybridizations of DNA from placenta correlated well with each other and did not correlate with the nonamplified, SCOMP-amplified, or DOP-amplified DNA from UACC-812 cells. The DOP-amplified samples had the lowest correlation between each other and with the control samples.

### 1.9. FFPE Samples

First, both WGA methods were compared using three samples of microdissected breast cancer. Although nonsegmented profiles of both WGA methods showed similar correlation with corresponding controls (Table 2), there was a trend for a greater number of concordant spots within the SCOMP samples. The mean percentage of cDNA spots concordant with the controls for gain/loss assignment was 65.7, 68.5, and 71.7% for DOP, SCOMP, and reference duplicate control runs, respectively, where difference between the groups was not statistically significant. Among the cDNA clones assigned discordantly opposite to the controls as gain or loss, there were clones discordant recurrently in all samples within the groups of SCOMP, DOP, or duplicate control samples: 2.53, 1.56 and 1.61%, respectively (difference not significant). The difference with the Log2 values of the control or duplicate runs was similar between the groups: 0.25, 0.26, and 0.21, respectively (difference not significant). These recurrent spots were different within each group.

Since the purpose of aCGH is identification of regions of genomic gain or loss rather than values of individual cDNA clones, we aimed to assess regions of gains/losses identified by segmentation and moving averages (Figure 3). The median number of segments per sample was similar between the groups: 41, 40, 41, 37 for all, control, SCOMP, and DOP samples, respectively, where the median number of segments of gain was 20, 25, 20, 20, and segments of loss 20, 15, 21, 17. There was 42.5% genome length concordance between segments identified in DOP and corresponding nonamplified controls, which were significantly lower than the 78.6% reference length concordance (reference: duplicate runs of nonamplified samples, *P* < 0.01, Mann-Whitney). For the same FFPE samples, SCOMP-amplified DNA showed segmentation patterns similar to the nonamplified controls with a concordance rate of 68.5%, which was not significantly different from the reference runs (Table 2). A similar advantage of the SCOMP method was observed by comparing peaks of genomic alterations: SCOMP samples had 84.4% concordant peaks compared to 72.2% of DOP-amplified samples, where the latter was significantly lower than the reference concordance rate.

To better assess the preferred WGA amplification method, we added more samples to the SCOMP group. Overall, 67.8% of detected segments and 84.4% of peaks identified in SCOMP amplified samples were concordant with those of nonamplified controls. 

## 2. Discussion 

Our aim was to compare and validate whole genome amplification methods for aCGH of microdissected FFPE tissue. SCOMP was particularly promising because it has been successfully used for analysis of single cells isolated from a breast cancer cell line [59], disseminated tumour cells in minimal residual cancer in the bone marrow [67], DNA extracted from archival material [41], and circulating melanoma cells [61]. In all cases there was low amount of genomic DNA available for study. DOP-PCR on the other hand is a well-established, technically straightforward method that is widely accepted as a method of WGA. DOP-PCR-generated DNA has been used for many applications, including aCGH [68, 69]. In both of these publications genomic BAC arrays were used, which have lower resolution than other aCGH modalities. Previously, Stoecklein et al. [41] had shown that SCOMP was preferable to DOP-PCR for use with formalin-fixed samples; however, it was only validated using chromosomal CGH. We compared the techniques and tested further the better performing method by a high-resolution aCGH. 

To initially test the methods, we used better-quality DNA extracted from fresh samples and found SCOMP superior to DOP-PCR (Figure 1). To validate the aCGH platform before further analysis, we used quantitative PCR (Q-PCR) and tested 22 genes in the three groups of data—nonamplified, SCOMP-amplified, and DOP-amplified. The highest agreement was in the nonamplified and SCOMP-amplified datasets (Table 1).

Our next step was to compare the genomic profiles generated by aCGH. The profiles of the samples amplified by both techniques had similar correlation with the controls; however, there was a trend for the DOP group to have a higher percentage of discordant individual spots. Only a small proportion of the discordant spots were observed repeatedly within the samples of each group, which we interpreted as indication of the artifacts being predominantly random and not specific for cDNA clone sequence, spot position within the array, or other factors specific for amplification method or the microarray platform. After reliability of the aCGH platform and amplification techniques were evaluated, we proceeded to experiments with FFPE specimens. In these experiments, aCGH analysis showed consistently better performance of the SCOMP technique compared to DOP-PCR.

Since aCGH has been successfully used in breast cancer research to characterize breast cancer cell lines and identify regions of common genomic alterations in different cancer subtypes [65, 70], we used current approaches of aCGH analysis to study the effect of WGA on accurate detection of alterations. The segmentation algorithm we used has been successfully employed in multiple aCGH studies [71–73] and is becoming a routine tool for denoising aCGH data from clinical samples. Additionally, we complemented segmentation analysis by identifying peaks of genomic alterations. To avoid biases we intentionally did not use cut-offs to filter segments or peaks by the degree of significance. SCOMP showed higher fidelity and allowed identification of the genomic alterations detected in nonamplified samples by both segmentation and moving average analyses at rates close to the reference values. The difference between median rates of the SCOMP and the reference group was 11% and 10% for the segmentation and moving average analyses, respectively. Although they may serve as estimates of artifacts introduced by SCOMP amplification, the differences were not statistically significant. The DOP method showed significant alterations of the profiles introducing 36% and 22% of discordant segmentation and peak identification over the reference values (Table 2). Our concordance rates are representative of the techniques, and the actual rates are expected to vary with quality of samples and aCGH platforms used. Combined, our results show that of the methods tested SCOMP is the most suitable method for WGA of FFPE tissues and delivers results similar to nonamplified samples.

Understanding the genomic characteristics and evolution of breast cancer lesions is a necessary step to answer many of the questions posed in the clinical setting, including which lesions are more likely to develop local recurrence or metastasis and therefore who would benefit from adjuvant therapy. Unfortunately, the lesions of interest are usually small and the availability of genomic DNA for research extremely limited. Use of existing breast archival FFPE material is optimized by microdissecting samples to obtain homogeneous histologically defined cell populations from small-volume lesions. WGA cannot be avoided in these settings, and our data show that SCOMP has the potential to be an invaluable tool for breast cancer research.

## Figures and Tables

**Table 1:** Quantitative real-time PCR results compared with array CGH.

cDNA clone	Gene	Location	Gene relative quantity by Q-PCR	Copy number by aCGH		
				Control	SCOMP	DOP
H17813	TOP2A	17q21	5.86	++	+++	N
H59203	CDC6	17q21.3	2.87	+++	++++	N
H23044	TEM7	17q21.1	2.45	++	+++	+
H64260	PRKAG2	7q35	2.23	N	N	N
H18802	Link-GEFII	17q21.1	2.19	++++	++++	+
H46384	PRO2521	17q21.1	2.18	++++	++	N
T85025			1.97	++++	++++	N
H29706	GPC5	13q32	1.79	+++	++++	N
H59714	AK2	1p34	1.58	++	+++	N
321749			1.55	+	+++	N
H20867	PCDH9	13q14.3	1.46	+++	++++	N
H93272	CPM	12q15	1.42	+	+	N
AA011584			1.38	+	++	+
W24419			1.36	+++	+	N
H85791			1.32		+ + +	+
H01255	CLN5	13q21.1	1.22	+++	++	+
H62028	DYRK3	1q32	1.18	+	+	+
H14685	PTK2	8q24	1.06	N	N	N
R06520	PRKCBP1	20q13.12	0.98	N	N	N
R24935	CDA08	16q11.2	0.76	−	N	N
H46055	KIAA0725	8p11.21	0.75	+	N	N
H53288	BAG4	8p11.21	0.71	N	N	N

“−” Ratio less than 0.7, *N* = 0.7 − 1.3, “+” ratio greater than 1.3.

“++” Ratio greater than 2, “+++” ratio greater than 3, “++++” Ratio greater than 5.

**Table 2:** Assessment of fidelity of WGA of DNA extracted from breast cancer FFPE tissue. Amplification was performed by two methods: DOP and SCOMP. aCGH profiles of pre- (control) and post-amplification DNA samples were compared by correlation of raw aCGH data, segmentation and peaks of genomic alterations (means of three independent experiments).

	DOP versus control	SCOMP versus control	SCOMP versus control*	Control repeats
Correlation of nonsegmented profiles (Pearson)	*r* = 0.41 (<0.01)	*r* = 0.47 (<0.01)	*r* = 0.43 (<0.01)	*r* = 0.72
Gain/loss concordance of segmentation (% of genome length)	42.5% (<0.01)	68.5% (N/S)	67.8% (N/S)	78.6%
Peaks of amplified samples concordant to controls (% of all peaks)	72.2% (<0.01)	84.0% (N/S)	84.4% (N/S)	94.2%

Third column (*) represents the extended set of 6 SCOMP amplified samples. *P* values in brackets are calculated for difference with control repeats by two-tailed exact Mann-Whitney *U* test; N/S: not significant.
